# Application potential of chicken DNA chip in domestic pigeon species – Preliminary results

**DOI:** 10.1016/j.sjbs.2023.103594

**Published:** 2023-02-11

**Authors:** Katalin Balog, Alexandru Eugeniu Mizeranschi, George Wanjala, Bíborka Sipos, Szilvia Kusza, Zoltán Bagi

**Affiliations:** a University of Debrecen, Doctoral School of Animal Science, Böszörményi út 138, 4032, Debrecen, Hungary; b Centre for Agricultural Genomics and Biotechnology, Faculty of Agricultural and Food Sciences and Environmental Management, University of Debrecen, 4002 Debrecen, Hungary; c Research and Development Station for Bovine, Bodrogului 32, Arad 310059, Romania; d University of Debrecen, Faculty of Agricultural and Food Sciences and Environmental Management, Böszörményi út 138, 4032, Debrecen, Hungary

**Keywords:** DNA chip, SNP-array, Squab pigeon, Racing pigeon, Illumina, Genotyping

## Abstract

Introducing the SNP technology to pigeon breeding will enhance the competitiveness of a sector that produces one of the healthiest and best quality meats. The present study aimed to test the applicability of the Illumina Chicken_50K_CobbCons array on 24 domestic pigeon individuals from the Mirthys hybrids and Racing pigeon breeds. A total of 53,313 SNPs were genotyped. Principal component analysis shows a significant overlap between the two groups. The chip performed poorly in this data set, with a call rate per sample of 0.474 (49%). The low call rate was likely due to an increase in the evolutionary distance. A total of 356 SNPs were retained after a relatively strict quality control. We have demonstrated that it is technically feasible to use a chicken microarray chip on pigeon samples. Presumably, with a larger sample size and by assigning phenotypic data, efficiency would be improved, allowing more thorough analyses, such as genome-wide association studies.

## 1 Introduction

The domestic pigeon (*Columba livia domestica*) has always been a popular model species for scientific research because of its richness of form, colouration and behaviour (Darwin, 1859, Hsu et al., 2017, Peñuela et al., 2019, Sarker et al., 2019, Wimsatt et al., 2020, Yang et al., 2021). Only the European Association of Poultry, Pigeon and Rabbit Breeders officially registers more than 1,100 domestic pigeon breeds (Entente Européenne d’ Áviculture et de Cuniculture, 2018), but the real number of pigeon breeds can certainly be higher, because there are numerous non-federally registered local breeds in Europe and the other parts of the world. Pigeon breeds can be classified into groups based on the purpose of utilization, which can be the following: racing pigeons, fancy pigeons and squab pigeon. The common characteristic of the breeds belonging to the racing pigeon category is sport flying, the group of fancy pigeons consist of special breeds, these two breed groups do not generate profit from an economic point of view, the breeders keep them as a hobby. It is worth mentioning an important similarity between racing pigeons and squab pigeons; the nice meat shapes and muscularity are expected in both types, this justified the joint examination of this type of the two groups (Appendix Fig. S1). Pigeons are a good quality, high biological value meat source with high biological value and a high recoverable meat ratio (70%) (Omojola et al., 2012), and appear on the market as a special quality, premium product (Bagi and Kusza, 2014, Jilly, 2018). Pigeon racing is popular and economically significant in many countries around the world (Proskura et al., 2014, Dybus et al., 2018, Dybus et al., 2020, Chang et al., 2021). The appearance of a group of customers with special needs and/or awareness is also increasingly important worldwide, those who want to consume more easily digestible types of meat, or those who take into account the ecological footprint of meat production (e.g. water demand) or the naturalness of production (absence of GMOs, hormones, antibiotics). In the same way, various diets affect an ever wider segment of society, but the number of customers who are different from the usual and/or looking for high quality is also constantly growing (Osman et al., 2014). Pigeon hybrids can be ideal for meeting these needs. Therefore all novel scientific results concerning pigeons attract considerable interest and are rapidly utilized.

Previously, various genetic markers have been successfully used to explore genetic diversity and phylogenetic relationships, and to identify polymorphisms associated with competitive performance and meat production in the domestic pigeon, which has been effective in expanding our knowledge of the species (Bigi et al., 2016, Boer et al., 2019, Dementieva et al., 2021, Dybus et al., 2006, Dybus et al., 2018, Dybus et al., 2020, Jacob et al., 2015, Boer et al., 2019, Jędrzejczak-Silicka et al., 2019, Lee et al., 2007, Pacheco et al., 2020, Proskura et al., 2015, Ramadan et al., 2018, Stringham et al., 2012, Proskura et al., 2014, Proskura et al., 2015). In contrast to the more advanced technologies and methods (e.g., microarrays, next-generation sequencing, transcriptome sequencing, genome-wide association studies), these provide only a limited amount of data at higher unit cost. Genetic characterization of animal breeds is usually carried out with neutral markers, such as microsatellite analysis, but SNP panels help to examine the genome at a higher resolution. SNP chips can be used in a variety of genomic studies, including inference of population history, structure, and admixture, estimation of effective population size, QTL mapping strategies, and genome-wide association studies and genomic selection. It also allows the exploration of the degree of genomic variation and linkage disequilibrium (LD) between breeds. Because SNPs only focus on genetic sequences, they reduce evaluation time and cost. Compared with traditional approaches, SNP genotyping techniques provide an informative genetic background, improve breeding prediction accuracy and breeding quality on the farm (Chang et al., 2021, Huang et al., 2015, Muñoz et al., 2019, Gärke et al., 2011).

Due to the importance of the domestic pigeon, there is a great need for more detailed phylogenetic, diversity and genome association studies (in connection with meat and racing performance traits), for which SNP-based genotyping can be an universal tool. These days the use of genome- and marker-based selection supported by modern genomic technology in studies on various domestic animals (e.g., cattle, sheep, chicken, etc.) is very common (Hillier et al., 2004;Ibtisham et al., 2017; Fleming et al., 2016). The technology employing DNA microarrays (or SNP chips) with different densities is a highly efficient method for the comparison of genomes, the detection of SNPs, as well as for studying molecular variance, population structure, and genealogy. It yields much more information than earlier methods at lower specific cost. The adaptation of DNA microarray technology to pigeons would increase the information available for analysis in many areas, such as genomic diversity and phylogenetic studies, and improve selection methods in pigeon breeding.

As far as we know, SNP-based chip technology has not been attempted in the domestic pigeon species, and studies on gene polymorphisms are not widely extensive either.

Since there is no commercially available SNP chip specialized for the domestic pigeon, SNP chips developed for other domestic animal taxa appear to be the best adaptable alternatives. Nowadays, to investigate ecologically important traits in certain wildlife populations, this type of screening has been initiated in several species of songbirds, for example, in the case of the great tit (*Parus major*), several improvements have been made to the existing SNP chip, demonstrating the increasing use of this technology in evolutionary and ecological research (Van Bers et al., 2012, Kim et al., 2018). There have been several examples of successful (although less efficient) applications of species-specific chips to closely related species, for example, using the dog (*Canis familiaris*) chip to wolf (*Canis lupus*) (Harmoinen et al., 2021) and golden jackal (*Canis aureus*) (Pollinger et al., 2011), the pig (*Sus domesticus*) chip to wild boar (*Sus scrofa*) (Herrero-Medrano et al., 2013) the cattle (*Bos taurus*) chip to European (*Bison bonasus*) or American bison (*Bison bison*) (Pertoldi et al., 2010), and the chicken (*Gallus domesticus*) chip to various forms of grouse (Tetraoninae) (Minias et al., 2019). Commercial arrays developed for domesticated species have been used mostly to study wild species, but domestication and selection can make a significant difference. Miller et al. (2012) showed an exponential decrease in the retention of polymorphisms, and a linear decrease in call rate was also observed (∼1.5 % per million years) with increasing evolutionary distance. The chicken and pigeon lineages diverged more than 85 million years ago (Claramunt and Cracraft, 2015), which is quite significant. However, in this case both species are domesticated, and it is important to note that human selection often takes place in a similar environment and involves similar criteria. Carcass- and meat-related trait loci may be conserved among different species (Debus et al., 2001, Barendse, 2009, Zhao et al., 2020). This is also a logical conclusion due to the biological functions of skeletal muscle and the similar selection goals of domestic animals. This hypothesis is supported by the fact that successful cross-amplification of chicken microsatellites in domestic pigeons has already been reported (Sathyakumar, 2013). Based on this we tried to adapt the Illumina Chicken_50K_CobbCons array, developed for the closest species (chicken) to the domestic pigeon. The questions addressed were: (1) What is the efficiency of using the SNP chip developed for chickens in domestic pigeons? (2) Following necessary quality control screening, how many SNPs remain suitable for carrying out statistical tests/evaluations?

If successful, this approach could also be used to improve practical selection programmes. In addition, it could pave the way for more in-depth research into related species (order Columbiformes) using more modern methodologies.

## 2 Materials and methods

### 2.1 Illumina Chicken_50K_CobbCons array

Two important lines, the broiler and the laying line were included in developing this array account. In order to create the chip, additional SNPs were identified with other sequences in the chicken genome assembly. The SNP chip contains a total of 57,636 SNPs (Groenen et al., 2011) and these are also segregated in chicken populations. The SNP identification procedure appears to be highly reliable, with an overall validity rate of 94 % for SNPs on the chip. The high reliability of the chip is also due to the selection of SNPs with high MAF values. 328 SNPs from 454 sequence contigs of the chicken genome were mapped. During the development of the chip, the SNPs were selected based on a certain priority score, for which criteria such as validation, Illumina design score, MAF information in both broilers and eggs were taken into account.

### 2.2 Animal samples

A total of 24 blood samples (1 ml/animal) were collected from domestic pigeons (*Columba livia domestica*). Twelve of these were Mirthys hybrids, a genotype optimized for meat production. Another 12 pigeons were Racing Pigeon from racing lofts (Appendix Fig. S1). The study was carried out in accordance with the local ethics committee’s guidelines of the University of Debrecen.

### 2.3 SNP genotyping and data analysis

Individual sample genotyping was carried out using the Chicken 50K_CobbCons chip (Illumina, San Diego, CA, USA). Genotyping was performed by the Neogen Corporation (Lansing, Michigan, USA). Input files were created using RStudio software (Haneem et al., 2017). We included two groups based on the individual breed (squab and racing pigeons). Next, the SNPviewer 4.1.2. software (Biosearch Technologies, United Kingdom) was used to examine the call rate of alleles. We then checked the GenCall and Gentrain scores. It was very difficult to set appropriate parameters for quality control; we tried to keep as many variables as possible in the study. Since pigeons have 80 chromosomes, the set flag for autosomes was 40 (--chr-set 40), and in the case of birds, the sex-determining chromosome has a different name (ZZ, ZW), therefore we also set their acceptance (--allow-extra-chr). Subsequently, to avoid excluding all variants, we set the --geno 0.05 flag. The next flag we used removes all SNPs with minor allele frequencies<0.01. The --indep pairwise 50 5 0.5/ 50 5 0.9 flag values greater than 0.5/ 0.9 were excluded from the analysis. For the analyses, we used the argyle package and the PLINK ver. 1.9. (Chang et al., 2015, Morgan, 2015).

## 3 Results

A total of 53,313 SNPs were genotyped in the two pigeon breeds. Evaluation of the GenCall and GenTrain scores was then started. GenCall can be used to rank and filter out failed genotypes, DNAs, and/or loci. The GenCall score cutoffs are used to assess genotyping quality, with scores below 0.2 generally indicating failed genotypes, whereas scores above 0.7 generally indicate well-functioning genotypes (Edriss et al., 2013). In our case there were no scores below 0.2, therefore all were placed in the well-functioning category. In Table 1 and Table 2 we summarized the per-sample GenCall rate values for the two groups (squab and racing pigeon). The average call rate for all the samples was 0.474, whereas the average for the squab pigeon group was 0.501 and for the racing pigeons, 0.447.

**Table 1:** The GeneCall rate values for squab pigeons per-sample.

**DNA ID**	**Call rate**
334	0.501
358	0.384
370	0.456
371	0.430
385	0.473
388	0.465
392	0.593
411	0.761
412	0.368
417	0.451
420	0.572
499	0.562
**Mean**	0.501

**Table 2:** The GeneCall rate values for racing pigeons per-sample.

**DNA ID**	**Call rate**
533	0.557
534	0.576
535	0.443
536	0.452
537	0.433
539	0.381
541	0.408
542	0.500
543	0.345
544	0.424
545	0.396
546	0.448
**Mean**	0.447

Table 3 and Table 4 show the GC values at 50% and 10%, respectively, indicating the accuracy of genotyping in the loci. It can be seen from the tables that of the 53,313 cases, only an extremely small number, 11 and 21 loci remain after filtering at 0.96 and 0.97, respectively. Table 5 and Table 6 show the 50% and 10% GC score values with different thresholds. For the GenTrain score, out of 57,636 cases, 70 cases scored 0.95 or above, which are summarized in Appendix Table S1.

**Table 3:** The 50% GC values and their corresponding loci are 0.97 or above.

**Locus Name**	**50 % GC Score**
Gga_rs13639808	0.973
Gga_rs13812139	0.971
Gga_rs14169707	0.970
Gga_rs14645079	0.971
Gga_rs14955642	0.972
Gga_rs15156959	0.973
Gga_rs15355522	0.971
Gga_rs15446715	0.970
Gga_rs15696835	0.973
Gga_rs16250090	0.972
GGaluGA331730	0.972

**Table 4:** The 10% GC values and their corresponding loci are 0.96 or above.

**Locus Name**	**10 % GC Score**
Gga_rs13623776	0.962
Gga_rs13651288	0.961
Gga_rs14113315	0.964
Gga_rs14278292	0.963
Gga_rs14321447	0.967
Gga_rs14389188	0.968
Gga_rs14406330	0.969
Gga_rs14603968	0.962
Gga_rs14645079	0.971
Gga_rs14691722	0.961
Gga_rs14774295	0.965
Gga_rs15143506	0.964
Gga_rs15169386	0.963
Gga_rs15839686	0.961
Gga_rs15898329	0.963
Gga_rs15919667	0.960
Gga_rs16101593	0.963
Gga_rs16265898	0.962
Gga_rs16729144	0.960
GGaluGA221832	0.962
GGaluGA346860	0.963

**Table 5:** Distribution of 50% GC scores of marker genotypes over all loci.

**Tresholds**	**Number of loci**
0 < 0.15	11,559
0.15 < GC < 0.40	16,329
0.50 < GC < 0.60	3,317
0.70 < GC < 0.80	3,859
0.80 < GC < 0.90	4,991
0.90<	3,981

**Table 6:** Distribution of 10% GC scores of marker genotypes over all loci.

**Tresholds**	**Number of loci**
0 < 0.15	40,619
0.15 < GC < 0.40	7,453
0.50 < GC < 0.60	682
0.70 < GC < 0.80	546
0.80 < GC < 0.90	624
0.90<	389

Allele call rates were computed via SNPviewer, for a value of 49%. This low value was probably due to the low number of individuals and missing phenotypic values, and the call rate per sample was 0.474, which was also<50 %. We calculated the linkage disequilibrium (LD) value for each SNP, characterized by the correlation coefficient (R^2^). This value can vary between 0 and 1, with 0 indicating that in a pairwise comparison, the two alleles are inherited independently (i.e., they are “in equilibrium” with each other), whereas a value of 1 indicates complete linkage, i.e., the two alleles always occur together in the population. For all SNPs, 27% of our data set showed complete linkage, probably due to the origin of the two groups. The list of 356 SNPs remaining after quality control is summarized in Appendix Table S2. After quality control and R^2^ testing, we wanted to test the ld-based pruning for the 356 SNPs because of the linkage results. To do this, we used the indep-pairwise command from PLINK, which prunes SNPs based on variance inflation factor (VIF), and recursively removes those below the set value within a sliding window. This method is based on pairwise genotypic correlation. This test generates two SNP lists, a list of excised SNPs and another of retained SNPs. When a parameter of --50 5 0.5 was entered for the indep pairwise command, values greater than 0.5 were excluded from the analysis, and in this case 179 SNPs were excluded out of 356 SNPs. With a value of 0.9, 38 SNPs were removed. These values support the percentage of R^2^ values performed for all SNPs, as the percentage of SNPs with solid linkage is negligible compared to those with medium linkage. In Table 7 we summarized the R^2^ values of significant SNPs from the literature (Luo et al., 2014, Mignon-Grasteau et al., 2015, Bihan-Duval et al., 2018, Pampouille et al., 2018, Li and Li, 2019, Zhang et al., 2020).

**Table 7:** SNPs based on literature with R^2^ values.

**SNP**	**CHR_A**	**BP_A**	**SNP_A**	**CHR_B**	**BP_B**	**SNP_B**	**R^2^**
**Gga_rs14769351**	Z	51,758,183	Gga_rs13768836	Z	51,933,697	Gga_rs14769351	0.282
	Z	51,804,319	Gga_rs14769274	Z	51,933,697	Gga_rs14769351	0.317
	Z	51,838,052	GGaluGA353240	Z	51,933,697	Gga_rs14769351	0.259
	Z	51,861,645	Gga_rs14769311	Z	51,933,697	Gga_rs14769351	0.329
	Z	51,874,066	GGaluGA353255	Z	51,933,697	Gga_rs14769351	0.316
	Z	51,902,294	Gga_rs14769339	Z	51,933,697	Gga_rs14769351	0.326
	Z	51,933,697	Gga_rs14769351	Z	52,093,707	Gga_rs14769471	0.045
	Z	51,933,697	Gga_rs14769351	Z	52,110,787	GGaluGA353313	0.234
	Z	51,933,697	Gga_rs14769351	Z	52,127,726	GGaluGA353318	0.044
	Z	51,933,697	Gga_rs14769351	Z	52,149,216	Gga_rs14769506	0.258
**Gga_rs14902012**	1	151,513,250	GGaluGA050024	1	151,674,077	Gga_rs14902012	0.446
	1	151,541,208	Gga_rs13955386	1	151,674,077	Gga_rs14902012	1
	1	151,674,077	Gga_rs14902012	1	151,716,082	Gga_rs15460229	1
	1	151,674,077	Gga_rs14902012	1	151,723,704	Gga_rs13955548	0.568
	1	151,674,077	Gga_rs14902012	1	151,798,977	Gga_rs13955659	0.345
	1	151,674,077	Gga_rs14902012	1	151,846,128	Gga_rs15460398	0.349
	1	151,674,077	Gga_rs14902012	1	151,882,692	Gga_rs13955712	0.9
	1	151,674,077	Gga_rs14902012	1	151,895,690	Gga_rs13955716	0.323
	1	151,616,243	GGaluGA050037	1	151,674,077	Gga_rs14902012	0.742
**Gga_rs15060839**	2	8,366,215	Gga_rs14135744	2	8,567,871	Gga_rs15060839	0.415
	2	8,399,260	Gga_rs14135812	2	8,567,871	Gga_rs15060839	0.215
	2	8,427,707	Gga_rs14135839	2	8,567,871	Gga_rs15060839	0.228
	2	8,458,409	GGaluGA132838	2	8,567,871	Gga_rs15060839	1
	2	8,498,212	GGaluGA132855	2	8,567,871	Gga_rs15060839	0.511
	2	8,533,018	GGaluGA132861	2	8,567,871	Gga_rs15060839	0.612
	2	8,567,871	Gga_rs15060839	2	8,650,283	Gga_rs13536859	1
	2	8,567,871	Gga_rs15060839	2	8,677,097	GGaluGA132897	0.555
	2	8,567,871	Gga_rs15060839	2	8,716,749	Gga_rs13536888	0.538
	2	8,567,871	Gga_rs15060839	2	8,751,462	GGaluGA132922	0.200
**Gga_rs15990597**	Z	73,314,219	Gga_rs16683601	Z	73,541,821	Gga_rs15990597	0.285
	Z	73,335,087	Gga_rs14685517	Z	73,541,821	Gga_rs15990597	0.333
	Z	73,354,579	Gga_rs14685507	Z	73,541,821	Gga_rs15990597	0.217
	Z	73,389,952	Gga_rs14685494	Z	73,541,821	Gga_rs15990597	0.409
	Z	73,432,595	Gga_rs15991578	Z	73,541,821	Gga_rs15990597	0.831
	Z	73,456,840	Gga_rs14743561	Z	73,541,821	Gga_rs15990597	0.457
	Z	73,479,829	Gga_rs16087363	Z	73,541,821	Gga_rs15990597	0.269
	Z	73,541,821	Gga_rs15990597	Z	73,593,177	Gga_rs14684720	0.228
	Z	73,541,821	Gga_rs15990597	Z	73,638,063	Gga_rs15990713	0.727
	Z	73,541,821	Gga_rs15990597	Z	73,657,363	Gga_rs14684801	1
	Z	73,541,821	Gga_rs15990597	Z	73,680,011	Gga_rs14684826	0.731
	Z	73,541,821	Gga_rs15990597	Z	73,723,053	Gga_rs15990798	0.425
	Z	73,541,821	Gga_rs15990597	Z	73,743,296	Gga_rs14684876	0.439
**Gga_rs16650878**	8	30,476,893	Gga_rs15942756	8	30,630,313	Gga_rs16650878	0.587
	8	30,484,231	Gga_rs16650811	8	30,630,313	Gga_rs16650878	1
	8	30,516,115	Gga_rs14658627	8	30,630,313	Gga_rs16650878	0.765
	8	30,518,213	GGaluGA333545	8	30,630,313	Gga_rs16650878	0.533
**GGaluGA287132**	5	49,329,620	Gga_rs14543719	5	49,506,678	GGaluGA287132	0.272
	5	49,336,875	GGaluGA287110	5	49,506,678	GGaluGA287132	0.245
	5	49,357,471	GGaluGA287112	5	49,506,678	GGaluGA287132	1
	5	49,398,171	Gga_rs16505237	5	49,506,678	GGaluGA287132	1
	5	49,477,573	Gga_rs14543773	5	49,506,678	GGaluGA287132	1
	5	49,506,678	GGaluGA287132	5	49,529,646	Gga_rs16505363	1
	5	49,506,678	GGaluGA287132	5	49,588,594	Gga_rs14543844	0.272
	5	49,506,678	GGaluGA287132	5	49,609,623	GGaluGA287141	0.392
	5	49,506,678	GGaluGA287132	5	49,658,774	Gga_rs14543895	1
	5	49,506,678	GGaluGA287132	5	49,694,082	Gga_rs16505461	0.264
**GGaluGA263381**	4	67,253,998	GGaluGA263285	4	67,437,915	GGaluGA263381	0.583
	4	67,274,906	Gga_rs16426682	4	67,437,915	GGaluGA263381	0.463
	4	67,289,718	Gga_rs14483584	4	67,437,915	GGaluGA263381	0.597
	4	67,318,887	GGaluGA263331	4	67,437,915	GGaluGA263381	0.441
	4	67,369,879	GGaluGA263357	4	67,437,915	GGaluGA263381	0.600
	4	67,389,921	GGaluGA263363	4	67,437,915	GGaluGA263381	0.552
	4	67,427,107	Gga_rs16426865	4	67,437,915	GGaluGA263381	0.214
	4	67,437,915	GGaluGA263381	4	67,458,181	GGaluGA263389	0.814
	4	67,437,915	GGaluGA263381	4	67,491,463	GGaluGA263399	0.468
	4	67,437,915	GGaluGA263381	4	67,494,877	GGaluGA263401	0.432
	4	67,437,915	GGaluGA263381	4	67,590,939	GGaluGA263439	0.576
	4	67,437,915	GGaluGA263381	4	67,618,962	Gga_rs14483890	0.349
	4	67,437,915	GGaluGA263381	4	67,663,914	GGaluGA263464	1
	4	67,437,915	GGaluGA263381	4	67,678,721	GGaluGA263467	0.650
**GGaluGA273676**	5	8,625,056	Gga_rs14511113	5	8,758,944	GGaluGA273676	0.323
	5	8,758,944	GGaluGA273676	5	8,841,213	GGaluGA273721	0.290
	5	8,758,944	GGaluGA273676	5	8,914,272	Gga_rs16460497	1
	5	8,758,944	GGaluGA273676	5	8,922,412	GGaluGA273743	0.251

**Fig. 1:**
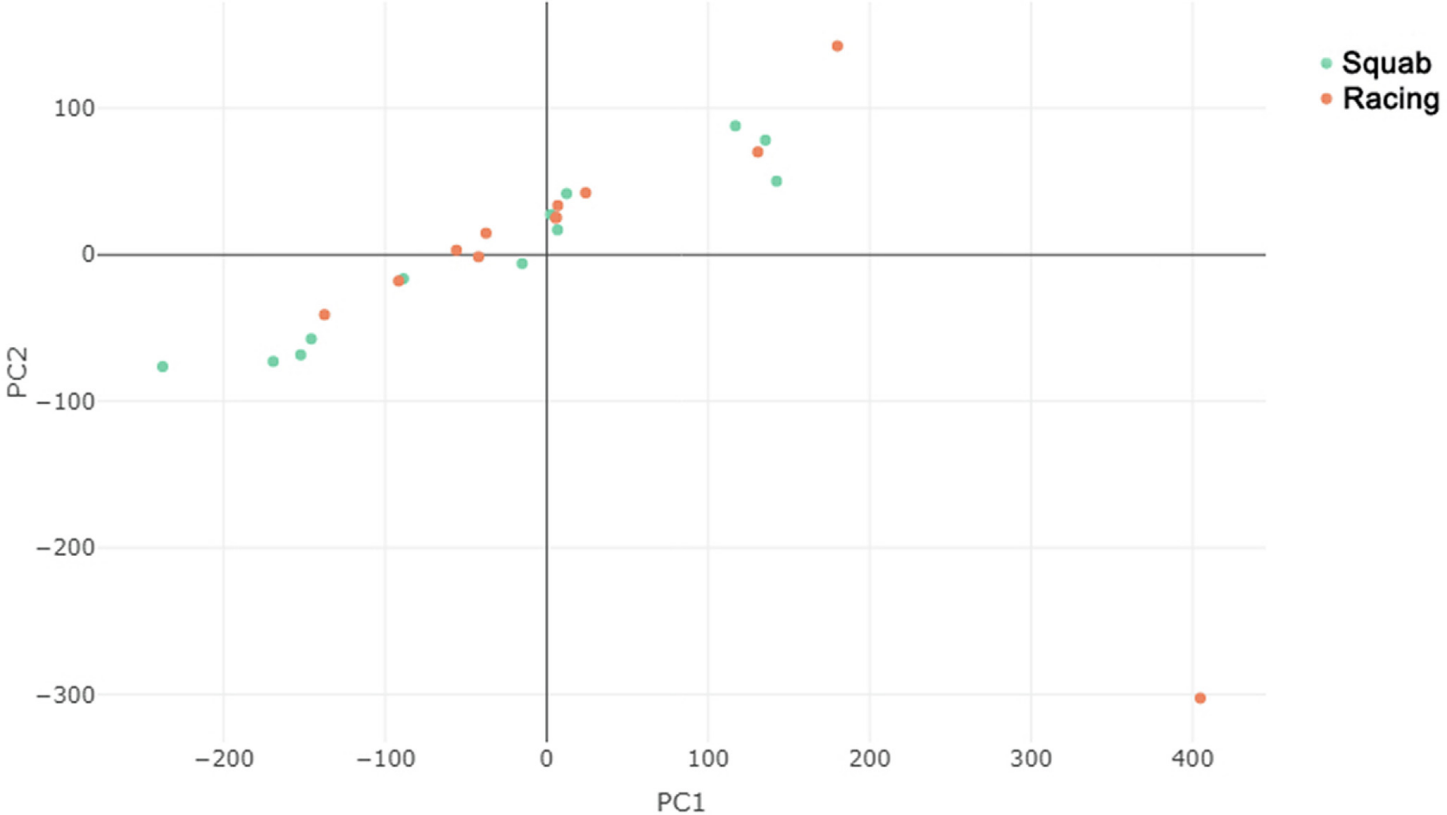
Clustering of two species (12 individuals per group) with Principal Component Analysis using autosomal loci.

Principal component analysis were performed to investigate the overlap between the two breeds. After stringent quality control (QC) conditions, 356 SNPs remained in the study and were used for principal component analysis (PCA) (Fig. 1).

As shown in Fig. 1, the two groups are not entirely separated; they overlap. The reason can be that the two pigeon types employed for different purposes still not to be significantly separated from each other on molecular genetic level. This can also be explained by the fact that muscles play an important role in both utilization types. One individual was an outlier in the squab pigeon group and two individuals in the racing pigeon group.

## 4 Discussion

Currently, commercially available DNA chips are species-specifically effective. They are also applicable with lower efficiency even in some taxonomically related or chromosomally similar species (Pertoldi et al., 2010, Hoffman et al., 2013, Minias et al., 2019, Abdelmanova et al., 2021, Fountain et al., 2021).

In 2021, Berry et al. quantified the rate of genotype and allele matching between single nucleotide polymorphism (SNP) genotypes differing in GenCall (GC) score on 771 cattle and 80 sheep samples. They found that the effect was larger and more consistent in the cattle population with more individuals than in the sheep population. In this study, we used thresholds (Oliphant et al., 2002) for GenCall, which put genotypes into the relatively well-functioning category. Based on the GenTrain measure (Luigi-Sierra et al., 2021), 5.29% of our data fell into the category of 0.5 or below, whereas 75% of the data were 0.8 or above. In fact, out of 57,636 cases, 70 scores of 0.95 or higher were obtained. Studies on phylogenetically divergent taxa can provide important clues to low call rates; in 2013, Hoffman et al. used a CanineHD BeadChip to genotype 24 Antarctic fur seals (*Arctocephalus gazella*). Seals and dogs diverged about 44 million years ago, resulting in a genotyping rate of 19.2%. The effectiveness of DNA chips has also been tested in non-model organisms, but closely related within species, in orangutans (*Pongo* spp.), chimpanzees (*Pan troglodytes*), and gorillas (*Gorilla* spp.) (Fountain et al., 2021). Using a specific microarray technique, the recovery rates of polymorphic SNPs were slightly higher than in their previous studies (4% in chimpanzees, 4% in gorillas, and 5% in orangutans). However, their high relative genotyping rates may be due to a relatively recent separation from the common ancestor (Fountain et al., 2021). In our case, the separation of pigeons and chickens occurred 85 million years ago (Claramunt and Cracraft, 2015), which, in addition to the low number of individuals and missing phenotypes, probably accounts for the low call rate (49%).

The reliability of genotyping is usually estimated with several measures, two of which are the 50% GC score and the 10% GC score. For each sample, this represents the 10th and 50th percentiles, respectively, of the distribution of GenCall scores across all called genotypes. At very high values, such as 0.96 and 0.97, 11 and 21 loci were detected. However, the smaller the value we used after filtering, the more loci remained in our data series, which suggests the success of the medium call rate.

SNPs were evaluated via the squared correlation value (R^2^) of the linkage disequilibrium. The design and interpretation of genetic association studies depend on the relationship between the genotyped variants and are often parameterized as the squared correlation or R^2^ measure of linkage disequilibrium between two loci (Wray, 2005). When designing association studies, selecting markers that are representative of the LD landscape of the region under study is common, such that the excluded markers are in high LD with one or a combination of the selected markers, for example, when studying associations with disease. A similar method can also be used to perform SNP selection, excluding markers with R^2^ values greater than a certain threshold with any of the selected markers (e.g., Carlson et al., 2004). This involves selecting SNPs based on their frequency to increase the likelihood of detecting an association with a nearby LD (Garner and Slatkin, 2003, Ohashi and Tokunaga, 2001). A similar method can also be used to perform SNP selection, excluding markers with an R^2^ greater than a certain threshold with any of the selected markers (e.g. Carlson et al, 2004). This involves selecting SNPs based on their frequency to increase the likelihood of detecting an association with a nearby square (Garner and Slatkin, 2003, Ohashi and Tokunaga, 2001). This method, which excludes SNPs only if the R^2^ with the retained SNP is greater than a certain t threshold, imposes stringent constraints on the allele frequencies of the excluded SNPs. For the 356 SNPs that remained after the quality check, we searched the NCBI dbSNP database (https://www.ncbi.nlm.nih.gov/snp/) and found 7 studies that examined 8 SNPs that were also detected in our dataset (Li et al., 2013, Luo et al., 2014, Mignon-Grasteau et al., 2015, Bihan-Duval et al., 2018, Pampouille et al., 2018, Li and Li, 2019, Zhang et al., 2020), and the literature found suggests that these SNPs are associated with meat production, feed utilization, and abdominal fat development in poultry.

Pairwise epistatic interaction was investigated using a 60 K single nucleotide polymorphism (SNP) chip in an 11th generation broiler chicken line differentially selected for abdominal fat content by Li et al. (2013). They performed network analysis and found that SNPs located in the middle of the subnetwork, Gga_rs14303341 and Gga_rs14988623, are important nodes in the development of abdominal fat (Jiang et al., 2014). In this study, the function of 5 genes in one of the subnetworks was significantly affected in the SNP Gga_rs14988623, which we also detected. The protein encoded by the FPGT gene is involved in an alternative pathway with a role in mammalian tissue formation (Makunin et al., 2016). TNNI3K is a cardiac-specific kinase that plays an important role in the cardiovascular system (Rabie et al., 2005). CRYZ is the major protein of the vertebrate eye lens (Graw, 2009). The protein encoded by the LHX8 gene is a member of the LIM homeobox protein family, which is involved in patterning and differentiation of various tissue types (Nakamura et al, 2008). In our study, the Gga_rs14988623 SNP on chromosome 13 also showed a high R^2^ value in all pairwise comparisons (Table 1), indicating high heritability, and a high GenTrain score (0.761).

A similar study was conducted by Zhang et al. in 2020 on chickens' carcass, growth and meat characteristics. For this study, lean and fat broiler chicken lines were selected and a total of 132 haplotypes were significantly associated with abdominal fat mass, among which the SNP Gga_rs15060839 on chromosome 2 was mentioned, which we also detected in our data. In this case, we also obtained a high GenTrain value (0.8208), but the R^2^ value was high in only one case (Table 7).

In 2014, Luo and colleagues investigated the major loci influencing the immune response to IBV, using 43,211 SNP markers to perform a genome-wide association study. This study identified 20 single-nucleotide polymorphisms that affect the levels of anti-IBV antibodies Patterson et al., 2006, Petit et al., 2017. These included the GGaluGA287132 SNP located on chromosome 5. It was also located on chromosome 5 in our pigeon samples, but in our case it showed a low R^2^ value in pairwise comparisons (Table 7), but the GenTrain score was very high (0.923) (Table S1).

In 820 meat chickens, birds were studied for growth, feed intake and feed conversion ratio, breast and abdominal fat yield, and digestive tract anatomy (Mignon-Grasteau et al., 2015). 16 QTLs were detected for feed intake, 13 for feed efficiency, 49 for anatomy-related traits, 7 for growth, 6 for body composition and 10 for selection. Among the QTL, several QTL were found in the same position for traits that are very similar but occur at different ages. Such traits were feed intake, body weight and development of digestive tract anatomy. However, in this case, as only two SNPs were detected that were quite close to each other, the accuracy of localisation on this chromosome was poor. In this study, the SNP Gga_rs14769351 was on chromosome ZW, which we also detected among our samples. This SNP did not show a high R^2^ value in our case (Table 7), but its GenTrain value was very high (0.898) (Table S1).

SNPs and QTL regions associated with meat quality traits were investigated in two different lines of chickens with meat defects (Pampouille et al., 2018). Several candidate genes involved in muscle metabolism and structure, as well as muscle dystrophies were identified. More than one third of the detected SNPs were located on GGA4 and two QTL regions (QTL3, QTL4) were identified. The first one (QTL3) was determined by a unique SNP (GGaluGA263381) that regulates both PMY and BMY (Markljung et al., 2012, Pampouille et al., 2018). We detected this SNP on chromosome 4 in pigeons, the R^2^ value was variable when tested, however the GenTrain value was high (0.921) (Table S1).

The SNP Gga_rs14902012 was also detected in pigeon samples, which was tested for pyloric pH by Bihan-Duval et al., 2018. In pairwise comparisons, this SNP occurred in 2 cases, one with a low value (0.446) and the other with the highest value (1), (Table 7) with a high GenTrain value (0.893) (Table S1).

SNP GgaluGA273676 is found in pigeons and has been studied in the past in relation to body weight in male birds (Li and Li, 2019). The R^2^ values in our case were low, with one case with a maximum value and a high GenTrain value (0.833).

All of the 8 SNPs found had a high GenTrain value in the pigeons, and from the R^2^ values it can also be concluded that these SNPs are inherited with relatively high reliability. In the future, during our investigations involving phenotypic data, these SNPs linked to meat quality could be targeted, and it can also be determined, at the individual level, in pigeons of which utilization type they occur in.

## 5 Conclusion

Despite the small number of elements and the lack of phenotypic data, SNPs that remained in the study after quality control could be associated with meat quality. We expected relatively weak genotyping values, which can be related to the low number of individuals and early evolutionary segregation. We found that the DNA chip developed for chickens can be used for genotyping domestic pigeons.

In the future, it would be worthwhile to conduct the experiment with biometric data (e.g. body weight, girth, breast bon length, height) during which many additional analyzes could be included. Therefore, we would like to record the body weight, body circumference and sternum length of the pigeons, as well as to examine the function of the already identified SNPs and possibly identify new ones. A correlation study could be conducted between gene polymorphisms and biometric data looking for significance, and allele and genotype frequencies, allele frequency variences and Hardy-Weinberg distribution could be examined per locus. Furthermore, we would like to find an answer to whether there is a difference between the genetic backgrounds of meat pigeons and homing pigeons. Also, we plan to conduct GWAS studies with a larger sample size and more breeds.

## 6 Data and model availability statement

None of the data were deposited in an official repository. The data presented in this study are available on request from the corresponding author.

## Funding

Supported by the ÚNKP-21–4 New National Excellence Program of the Ministry for Innovation and Technology from the source of the National Research, Development and Innovation Fund.

## Declaration of Competing Interest

The authors declare that they have no known competing financial interests or personal relationships that could have appeared to influence the work reported in this paper.

## References

[b0005] Abdelmanova A.S., Dotsev A.V., Romanov M.N., Stanishevskaya O.I., Gladyr E.A., Rodionov A.N., Vetokh A.N., Volkova N.A., Fedorova E.S., Gusev I.V. (2021). Unveiling comparative genomic trajectories of selection and key candidate genes in egg-type russian white and meat-type white cornish chickens. Biology.

[b0010] Bagi, Z., Kusza, S. Values of Hungarian pigeon breeding. 2014. AAD. (57), 9–14. https://doi.org/10.34101/actaagrar/57/1952

[b0015] Barendse, W. 2009. Genetic-based diagnostic tools for predicting meat quality. In: Improving the sensory and nutritional quality of fresh meat, CSIRO Livestock Industries, Australia. 292–317. https://doi.org/10.1533/9781845695439.2.292.

[b0020] Berry D.P., Dunne F.L., Evans R.D., McDermott K., O’Brien A.C. (2021). Concordance rate in cattle and sheep between genotypes differing in Illumina GenCall quality score. Anim. Genet..

[b0025] Bigi D., Mucci N., Mengoni C., Baldaccini E.N., Randi E. (2016). Genetic investigation of Italian domestic pigeons increases knowledge about the long-bred history of *Columba livia* (Aves: Columbidae). Ital. J. Zool..

[b0030] Bihan-Duval E.L., Hennequet-Antier C., Berri C., Beauclercq S.A., Bourin M.C., Boulay M., Boitard S. (2018). Identification of genomic regions and candidate genes for chicken meat ultimate pH by combined detection of selection signatures and QTL. BMC Genom..

[b0035] Boer E.F., Van Hollebeke H.F., Park S., Infante C.R., Menke D.B., Shapiro M.D. (2019). Pigeon foot feathering reveals conserved limb identity networks. Dev. Biol..

[b0040] Carlson C.S., Eberle M.A., Rieder M.J., Yi Q., Kruglyak L., Nickerson D.A. (2004). Selecting a maximally informative set of single-nucleotide polymorphisms for association analyses using linkage disequilibrium. AJHG.

[b0045] Chang C.C., Chow C.C., Tellier L.C., Vattikuti S., Purcell S.M., Lee J.J. (2015). Second-generation PLINK: rising to the challenge of larger and richer datasets. GigaScience.

[b0050] Chang C.C., Silva B.B.I., Huang H.Y., Tsai C.Y., Flores R.J.D., Tayo L.L., Yang J.L. (2021). Development and validation of KASP assays for the genotyping of racing performance-associated single nucleotide polymorphisms in pigeons. Genes.

[b0055] Claramunt S., Cracraft J. (2015). A new time tree reveals Earth history’s imprint on the evolution of modern birds. Sci. Adv..

[b0060] Darwin, C. 1859. On the Origin of Species by Means of Natural Selection John Murray, London, pp. 336.

[b0065] Debus N., Dutour A., Vuaroqueaux V., Oliver C., Ouafik L.H. (2001). The ovine somatostatin receptor subtype 1 (osst1): partial cloning and tissue distribution. Domest. Anim. Endocrinol..

[b0070] Dementieva N.V., Mitrofanova O.V., Dysin A.P., Kudinov A.A., Stanishevskaya O.I., Larkina T.A., Plemyashov K.V., Griffin D.K., Romanov M.N., Smaragdov M.G. (2021). Assessing the effects of rare alleles and linkage disequilibrium on estimates of genetic diversity in the chicken populations. Animal.

[b0075] Dybus A., Pijanka J., Cheng Y.H., Sheen F., Grzesiak W., Muszyńska M. (2006). Polymorphism within the LDHA gene in the homing and non-homing pigeons. J. Appl. Genet..

[b0080] Dybus A., Proskura W.S., Pawlina E., Nowak B. (2018). Associations between polymorphisms in the myostatin, αA-globin and lactate dehydrogenase B genes and racing performance in homing pigeons. Vet. Med..

[b0085] Dybus A., Yu Y.H., Proskura W., Lanckriet R., Cheng Y.H. (2020). Association of sequence variants in the CKM (Creatine Kinase, M-Type) gene with racing performance of homing pigeons. Russ. J. Genet..

[b0090] Edriss V., Guldbrandtsen B., Lund M.S., Su G. (2013). Criteria of GenCall score to edit marker data and methods to handle missing markers have an influence on accuracy of genomic predictions. Arch. Anim. Breed..

[b0095] Entente Européenne d’ Áviculture et de Cuniculture, 2018. List of the breeds of fancy pigeons. https://www.entente-ee.com/wp-content/uploads/1-Liste-der-Rassetauben-ELRT-01-10-2018.pdf (accessed 29 August 2022)

[bib381] Fleming D.S., Koltes J.E., Markey A.D., Schmidt C.J., Ashwell C., Rothschild M.F., Persia M.E., Reecy J.M., Lamont S.J. (2016). Genomic analysis of Ugandan and Rwandan chicken ecotypes using a 600 k genotyping array. BMC Gen.

[b0100] Fountain E.D., Zhou L.-C., Karklus A., Liu Q.-X., Meyers J., Fontanilla I.K.C., Rafael E.F., Yu J.-Y., Zhang Q., Zhu X.-L., Pei E.-L., Yuan Y.-H., Banes G.L. (2021). Cross-species application of Illumina iScan microarrays for cost-effective, high-throughput SNP discovery. Front. Ecol. Evol..

[b0105] Gärke, C., Ytournel, F., Bed’hom, B., Gut, I., Lathrop, M., Weigend, S., Simianer, H. 2011. Comparison of SNPs and microsatellites for assessing the genetic structure of chicken populations. Anim. Genet. 43(4), 419–428. https://doi.org/10.1111/j.1365-2052.2011.02284.x 10.1111/j.1365-2052.2011.02284.x 22497629

[b0110] Garner C., Slatkin M. (2003). On selecting markers for association studies: Patterns of linkage disequilibrium between two and three diallelic loci. Genet. Epidemiol..

[b0115] Graw J. (2009). Genetics of crystallins: cataract and beyond. Exp. Eye. Res..

[b0120] Groenen M.A., Megens H.-J., Zare Y., Warren W.C., Hillier L.W., Crooijmans R.P., Cheng H.H. (2011). The development and characterization of a 60K SNP chip for chicken. BMC Genom..

[b0130] Haneem F., Ali R., Kama N., Basri S. (2017). Descriptive analysis and text analysis in systematic literature review: a review of master data management. Int. Conf. Res. Innov. Information Syst. (ICRIIS).

[b0135] Harmoinen J., von Thaden A., Aspi J., Kvist L., Cocchiararo B., Jarausch A., Nowak C. (2021). Reliable wolf-dog hybrid detection in Europe using a reduced SNP panel developed for non-invasively collected samples. BMC Genom..

[b0140] Herrero-Medrano J.M., Megens H.J., Groenen M.A. (2013). Conservation genomic analysis of domestic and wild pig populations from the Iberian Peninsula. BMC Genet..

[b0145] Hillier L.W., Miller W., Birney E., Warren W., Hardison R.C., Ponting C.P., Bork P., Burt D.W., Groenen M.A.M., Delany M.E., Dodgson J.B., Chinwalla A.T., Cliften P.F., Clifton S.W., Delehaunty K.D., Fronick C., Fulton R.S., Graves T.A., Kremitzki C., Layman D., Magrini V., McPherson J.D., Miner T.L., Minx P., Nash W.E., Nhan M.N., Nelson J.O., Oddy L.G., Pohl C.S., Randall-Maher J. (2004). International chicken genome sequencing consortium, sequence and comparative analysis of the chicken genome provide unique perspectives on vertebrate evolution. Nature.

[b0150] Hoffman J.I., Thorne M.A.S., McEwing R., Forcada J., Ogden R. (2013). Cross-amplification and validation of snps conserved over 44 million years between seals and dogs. PLoS ONE.

[b0155] Hsu B.Y., Dijkstra C., Darras V.M., de Vries B., Groothuis T.G. (2017). Maternal thyroid hormones enhance hatching success but decrease nestling body mass in the rock pigeon (*Columba livia*). Gen. Comp. Endocrinol..

[b0160] Huang C.W., Lin Y.T., Ding S.T., Lo L.L., Wang P.H., Lin E.C., Lu Y.W. (2015). Efficient SNP discovery by combining microarray and lab-on-a-chip data for animal breeding and selection. Microarrays.

[b0165] Ibtisham F., Zhang L., Xiao M., An L., Ramzan M., Nawab A., Zhao Y., Li G., Xu Y. (2017). Genomic selection and its application in animal breeding. Thai. J. Vet. Med..

[b0170] Jacob G., Prévot-Julliard A.C., Baudry E. (2015). The geographic scale of genetic differentiation in the feral pigeon (*Columba livia*): implications for management. Biol. Invasions.

[b0175] Jędrzejczak-Silicka M., Dudaniec K., Dybus A. (2019). Association of alpha-a globin gene polymorphism with its expression level in racing pigeons. Acta. Sci. Pol. Zootech..

[b0180] Jiang J.H., Liu Y.F., Ke A.W., Gu F.M., Yu Y., Dai Z., Zhou J. (2014). Clinical significance of the ubiquitin ligase UBE3C in hepatocellular carcinoma revealed by exome sequencing. Hepatology.

[b0185] Jilly B. (2018). Development opportunities and profitability of private utility pigeon breeding in Hungary. Agricultural.

[b0190] Kim J.-M., Santure A.W., Barton H.J., Quinn J.L., Cole E.F., Visser M.E. (2018). A high-density SNP chip for genotyping great tit (*Parus major*) populations and its application to studying the genetic architecture of exploration behaviour. Mol. Ecol. Res..

[b0195] Lee J.C.I., Tsai L.C., Kuan Y.Y., Chien W.H., Chang K.T., Wu C.H., Hsieh H.M. (2007). Racing pigeon identification using STR and chromo-helicase DNA binding gene markers. Electrophoresis.

[b0200] Li F., Hu G., Zhang H., Wang S., Wang Z. (2013). Epistatic effects on abdominal fat content in chickens: results from a genome-wide SNP-SNP interaction analysis. PLoS ONE.

[b0205] Li F.-G., Li H. (2019). A time-dependent genome-wide SNP-SNP interaction analysis of chicken body weight. BMC Genom..

[b0210] Luigi-Sierra M.G., Casellas J., Martínez A., Vicente Delgado J., Fernández Álvarez J., Such F.X., Amills M. (2021). Markers with low GenTrain scores can generate spurious signals in genome-wide scans for transmission ratio distortion. Anim. Genet..

[b0215] Luo C., Qu H., Ma J., Wang J., Hu X., Li N., Shu D. (2014). A genome-wide association study identifies major loci affecting the immune response against infectious bronchitis virus in chicken. Infect. Genet. Evol..

[b0220] Makunin A.I., Kichigin I.G., Larkin D.M., O'Brien P.C.M., Ferguson-Smith M.A., Yang F., Trifonov V.A. (2016). Contrasting origin of B chromosomes in two cervids (Siberian roe deer and grey brocket deer) unravelled by chromosome-specific DNA sequencing. BMC Genom..

[b0225] Markljung E., Adamovic T., Cao J., Naji H., Kaiser S., Wester T., Nordenskjöld A. (2012). Novel mutations in the MNX1 gene in two families with Currarino syndrome and variable phenotype. Gene..

[b0230] Mignon-Grasteau S., Rideau N., Gabriel I., Chantry-Darmon C., Boscher M.-Y., Sellier N., Narcy A. (2015). Detection of QTL controlling feed efficiency and excretion in chickens fed a wheat-based diet. Genet. Sel..

[b0235] Miller J.M., Kijas J.W., Heaton M.P., McEwan J.C., Coltman D.W. (2012). Consistent divergence times and allele sharing measured from cross-species application of SNP chips developed for three domestic species. Mol. Ecol. Res..

[b0240] Minias P., Dunn P.O., Whittingham L.A., Johnson J.A., Oyler-McCance S.J. (2019). Evaluation of a Chicken 600K SNP genotyping array in non-model species of grouse. Sci. Rep..

[b0245] Morgan A.P. (2015). argyle: an R package for analysis of Illumina genotyping arrays. G3 Genes|Genomes|Genet..

[b0250] Muñoz M., Bozzi R., García-Casco J., Núñez Y., Ribani A., Franci O., Óvilo C. (2019). Genomic diversity, linkage disequilibrium and selection signatures in European local pig breeds assessed with a high density SNP chip. Sci. Rep..

[b0255] Nakamura Y., Fumitake U., Yasuhiro Y., Yusuke A., Tamao O., Kumiko T., Keijiro N., Hiroshi K., Takahiro T. (2008). A novel strategy for preservation of genetic resources in birds. Biol. Reprod..

[b0260] Ohashi J., Tokunaga K. (2001). The power of genome-wide association studies of complex disease genes: statistical limitations of indirect approaches using SNP markers. J. Hum. Genet..

[b0265] Oliphant, A., Barker, D.L., Stuelpnagel, J.R., Chee, M.S. 2002. BeadArray™ Technology: Enabling an Accurate, Cost-Effective Approach to High-Throughput Genotyping. Biotechniques. Suppl:56-8, 60–1. https://doi.org/10.2144/jun0207 12083399

[b0270] Omojola A.B., Isa M.A., Jibir M., Ajewole B.T., Garba S., Kassim O.R., Akinleye S.B. (2012). Carcass characteristics and meat attributes of pigeon (*Columbia livia*) as influenced by strain and sex. J. Anim. Sci. Adv..

[b0275] Osman K.M., Marouf S.H., Mehana O.A., Al Atfeehy N. (2014). *Salmonella enterica* serotypes isolated from squabs reveal multidrug resistance and a distinct pathogenicity gene repertoire. Rev. Sci. Tech. (International Office of Epizootics).

[b0280] Pacheco G., Van Grouw H., Shapiro M.D., Gilbert M.T.P., Vieira F.G. (2020). Darwin’s fancy revised: an updated understanding of the genomic constitution of pigeon breeds. GBE.

[b0285] Pampouille E., Berri C., Boitard S., Hennequet-Antier C., Beauclercq S.A., Godet E., Le Bihan-Duval E. (2018). Mapping QTL for white striping in relation to breast muscle yield and meat quality traits in broiler chickens. BMC Genom..

[b0290] Patterson N., Price A.L., Reich D. (2006). Population structure and eigenanalysis. PLoS Genet..

[b0295] Peñuela M., Rondón F., González R., Cárdenas H. (2019). Transcontinental genetic inference of urban pigeon populations using phenotypic markers. Avian. Biol. Res..

[b0300] Pertoldi C., Wójcik J.M., Tokarska M., Kawałko A., Kristensen T.N., Loeschcke V., Bendixen C. (2010). Genome variability in European and American bison detected using the BovineSNP50 BeadChip. Conserv..

[b0305] Petit F., Sears K.E., Ahituv N. (2017). Limb development: a paradigm of gene regulation. Nat. Rev. Genet..

[b0310] Pollinger J.P., Earl D.A., Knowles J.C., Boyko A.R., Parker H., Geffen E., Wayne R.K. (2011). A genome-wide perspective on the evolutionary history of enigmatic wolf-like canids. Gen. Res..

[b0315] Proskura, W.S., Cichoń, D., Grzesiak, W., Zaborski, D., Sell-Kubiak, E., Cheng, Y.H., Dybus, A. 2014. Single nucleotide polymorphism in the LDHA gene as a potential marker for the racing performance of pigeons. Poult. Sci. J. 0130237. https://doi.org/10.2141/jpsa.0130237

[b0320] Proskura, W.S., Dybus, A., Łukaszewicz, A., Hardziejewicz, E., Pawlina, E. 2015. The single nucleotide polymorphisms in lactate dehydrogenase-a (LDHA) and feather keratin (F-KER) genes and racing performance of domestic pigeon. Zeszyty Naukowe Uniwersytetu, Przyrodniczego we Wrocławiu-Biologia i Hodowla Zwierząt 76, 37–42

[b0325] Proskura W.S., Kustosz J., Dybus A., Lanckriet R. (2015). Polymorphism in dopamine receptor D4 gene is associated with pigeon racing performance. Anim. Genet..

[b0330] Rabie T.S.K.M., Crooijmans R.P.M.A., Bovenhuis H., Vereijken A.L.J., Veenendaal T., Poel J.J., Groenen M.A.M. (2005). Genetic mapping of quantitative trait loci affecting susceptibility in chicken to develop pulmonary hypertension syndrome. Anim. Genet..

[b0335] Ramadan S., Dawod A., El-Garhy O., Nowier A.M., Eltanany M., Inoue-Murayama M. (2018). Genetic characterization of 11 microsatellite loci in Egyptian pigeons (*Columba livia domestica*) and their cross-species amplification in other Columbidae populations. Vet. World.

[b0340] Sarker S., Das S., Ghorashi S.A., Forwood J.K., Raidal S.R. (2019). Pigeon circoviruses from feral pigeons in Australia demonstrate extensive recombination and genetic admixture with other circoviruses. Avian. Pathol..

[b0345] Sathyakumar S. (2013). Eighteen polymorphic microsatellites for domestic pigeon *Columba livia var. domestica* developed by cross species amplification of chicken markers. J. Genet..

[b0350] Stringham S.A., Mulroy E.E., Xing J., Record D., Guernsey M.W., Aldenhoven J.T., Shapiro M.D. (2012). Divergence, convergence, and the ancestry of feral populations in the domestic rock pigeon. Curr. Biol..

[b0355] Van Bers, N. E. M., Santure, A. W., Van Oers, K., DE Cauwer, I., Dibbits, B. W., Mateman, C., Slate, J. 2012. The design and cross-population application of a genome-wide SNP chip for the great tit *Parus major*. Mol. Ecol. Res., 12(4), 753–770. https://doi:10.1111/j.1755-0998.2012.03141.x 10.1111/j.1755-0998.2012.03141.x 22487530

[b0360] Wimsatt J., Pearce R., Steyn P.F., Vap L., Glover D.K. (2020). Comparative renal disposition of creatine, and technetium diagnostic contrast agents in the pigeon (*Columba livia*). JZWM.

[b0365] Wray N.R. (2005). Allele frequencies and the R^2^ measure of linkage disequilibrium: impact on design and interpretation of association studies. Twin. Res. Hum. Genet..

[b0370] Yang J., Gu J., Hu Y., Wang N., Gao J., Wang P. (2021). Molecular cloning and characterization of HSP60 gene in domestic pigeons (*Columba livia*) and differential expression patterns under temperature stress. Cell Stress Chaperones.

[b0375] Zhang H., Shen L.-Y., Xu Z.-C., Kramer L.M., Yu J.-Q., Zhang X.-Y., Li H. (2020). Haplotype-based genome-wide association studies for carcass and growth traits in chicken. Poult. Sci..

[b0380] Zhao C., Raza S.H.A., Khan R., Sabek A., Khan S., Ullah I., Zan L. (2020). Genetic variants in MYF5 affected growth traits and beef quality traits in Chinese Qinchuan cattle. Genomics.

