# Supplementary material for: Application potential of chicken DNA chip in domestic pigeon species – Preliminary results
Source: Saudi J Biol Sci. 2023 Feb 11;30(3):103594. doi: 10.1016/j.sjbs.2023.103594 (PMC9975693; doi:10.1016/j.sjbs.2023.103594)
Supplement: Supplementary data 1 [file mmc1.docx]

**Application potential of chicken DNA chip in domestic pigeon species – preliminary results**

Balog, K., Mizeranschi, A., Wanjala, G., Sipos, B., Kusza, Sz., Bagi, Z.

Saudi Journal of Biological Sciences


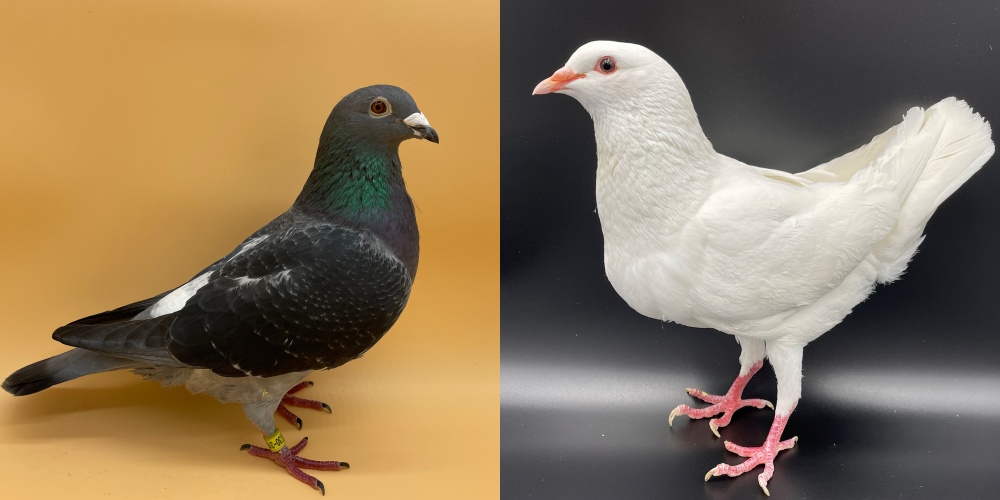


**Figure S1. The two utility types of pigeons used in the study; racing pigeon (left) and squab pigeon (right).**
