# Supplementary material for: Application potential of chicken DNA chip in domestic pigeon species – Preliminary results
Source: Saudi J Biol Sci. 2023 Feb 11;30(3):103594. doi: 10.1016/j.sjbs.2023.103594 (PMC9975693; doi:10.1016/j.sjbs.2023.103594)
Supplement: Supplementary data 2 [file mmc2.docx]

**Application potential of chicken DNA chip in domestic pigeon species – preliminary results**

Balog, K., Mizeranschi, A., Wanjala, G., Sipos, B., Kusza, Sz., Bagi, Z.

Saudi Journal of Biological Sciences

**Table S2. The list of 356 SNPs remaining after quality control**

GGaluGA000777

GGaluGA000920

GGaluGA001723

GGaluGA360407

GGaluGA360440

GGaluGA360465

GGaluGA003707

GGaluGA003770

Gga_rs16756550

Gga_rs13834498

Gga_rs13832954

Gga_rs13830900

Gga_rs3137220

GGaluGA006628

Gga_rs15205470

Gga_rs13837714

Gga_rs13837906

GGaluGA008639

Gga_rs15226336

Gga_rs14807453

Gga_rs15242113

Gga_rs15246972

Gga_rs14816659

Gga_rs13858221

Gga_rs13858322

GGaluGA017563

GGaluGA018020

GGaluGA018985

Gga_rs14830969

Gga_rs15306338

Gga_rs13996420

Gga_rs13996402

Gga_rs13888629

Gga_rs13646774

Gga_rs13906735

Gga_rs14862605

Gga_rs13909597

Gga_rs13685258

Gga_rs13685679

GGaluGA040636

Gga_rs13933311

Gga_rs13936113

GGaluGA044168

Gga_rs13940183

Gga_rs13944358

Gga_rs14894797

Gga_rs14897294

Gga_rs13949561

Gga_rs13713113

Gga_rs14902012

Gga_rs13956164

Gga_rs13956744

Gga_rs15473160

Gga_rs15480763

Gga_rs13959089

GGaluGA052917

Gga_rs13976345

GGaluGA056285

Gga_rs13978011

Gga_rs13980481

Gga_rs13981610

GGaluGA059094

GGaluGA059781

Gga_rs13987474

GGaluGA062679

GGaluGA062884

Gga_rs14131757

Gga_rs15059107

Gga_rs13536225

Gga_rs15060839

Gga_rs14137210

Gga_rs14138708

Gga_rs15891507

Gga_rs15063360

Gga_rs15892848

Gga_rs15907594

GGaluGA137110

Gga_rs14154677

Gga_rs15927561

GGaluGA141307

Gga_rs15084587

GGaluGA141823

GGaluGA141993

Gga_rs14167159

GGaluGA144250

Gga_rs15968949

Gga_rs15092643

Gga_rs14175008

Gga_rs15094411

GGaluGA145269

Gga_rs13674519

Gga_rs14182760

GGaluGA150403

Gga_rs16023012

GGaluGA152296

Gga_rs14203128

Gga_rs14203856

Gga_rs16039443

GGaluGA153863

Gga_rs13602234

Gga_rs16040849

Gga_rs14206942

Gga_rs16049986

Gga_rs13636957

Gga_rs14214752

Gga_rs16056916

Gga_rs14221592

Gga_rs16072368

Gga_rs15133512

Gga_rs15138955

GGaluGA162930

Gga_rs14234623

Gga_rs15143482

Gga_rs16020455

Gga_rs14240469

Gga_rs16133981

GGaluGA168333

GGaluGA168898

Gga_rs15156096

Gga_rs16139017

Gga_rs14253122

Gga_rs15156946

Gga_rs14255037

Gga_rs15158438

Gga_rs16153251

Gga_rs14263225

GGaluGA172303

Gga_rs14258876

Gga_rs14256306

GGaluGA210966

GGaluGA212610

Gga_rs16239463

GGaluGA213405

Gga_rs14331919

GGaluGA214212

Gga_rs14335265

Gga_rs15332830

Gga_rs16261830

Gga_rs16281869

GGaluGA223730

Gga_rs14365797

Gga_rs14368687

Gga_rs14373900

Gga_rs14375158

Gga_rs14375807

Gga_rs14376216

Gga_rs16300688

Gga_rs15391102

Gga_rs15403248

GGaluGA230674

Gga_rs14388780

Gga_rs14391034

GGaluGA234329

GGaluGA234360

GGaluGA234987

Gga_rs14410471

Gga_rs16343009

Gga_rs14414625

Gga_rs15484269

Gga_rs14424011

GGaluGA248467

GGaluGA249159

Gga_rs13576379

Gga_rs14437998

GGaluGA249741

Gga_rs14441946

Gga_rs14442279

GGaluGA252382

Gga_rs16396454

Gga_rs14479052

GGaluGA263381

GGaluGA263941

Gga_rs14487426

Gga_rs16432567

Gga_rs16434015

Gga_rs16435243

Gga_rs14707202

GGaluGA266814

Gga_rs16440139

Gga_rs13665391

Gga_rs15639283

Gga_rs15645991

Gga_rs15646226

Gga_rs16459730

GGaluGA273676

Gga_rs16464613

Gga_rs15661965

Gga_rs14514941

Gga_rs15662737

Gga_rs14515681

GGaluGA275640

Gga_rs15675732

GGaluGA277653

Gga_rs14521044

Gga_rs16691609

Gga_rs14522463

Gga_rs14531367

Gga_rs16492112

Gga_rs14538643

GGaluGA286448

GGaluGA287132

GGaluGA287195

Gga_rs15749506

Gga_rs16523775

Gga_rs14559429

Gga_rs14559787

Gga_rs15758345

Gga_rs16543585

Gga_rs14575093

Gga_rs14576983

Gga_rs14578130

Gga_rs14578549

GGaluGA300408

Gga_rs14579843

Gga_rs13568835

Gga_rs15795321

GGaluGA301379

Gga_rs14586853

Gga_rs14588627

Gga_rs14591343

Gga_rs14599802

Gga_rs14631747

Gga_rs16617752

GGaluGA325401

GGaluGA325666

Gga_rs13679994

GGaluGA327329

Gga_rs16641540

Gga_rs16649746

Gga_rs16650878

Gga_rs16652673

Gga_rs15967358

Gga_rs14670210

Gga_rs15957511

Gga_rs13763666

Gga_rs15971285

Gga_rs16674497

Gga_rs14678745

Gga_rs16676860

GGaluGA343290

GGaluGA344585

GGaluGA066412

Gga_rs13677696

Gga_rs14003248

Gga_rs15572342

GGaluGA068322

Gga_rs14004409

GGaluGA068485

Gga_rs15578499

Gga_rs14012168

Gga_rs13704694

Gga_rs15598849

Gga_rs14019663

Gga_rs14021152

Gga_rs15613566

Gga_rs15613810

Gga_rs15619325

Gga_rs15620221

GGaluGA078237

Gga_rs14026836

Gga_rs13700208

Gga_rs15627767

Gga_rs15627877

GGaluGA082666

Gga_rs14036286

Gga_rs14039714

GGaluGA085457

Gga_rs10731016

Gga_rs13612590

GGaluGA086740

Gga_rs14045323

Gga_rs14048983

Gga_rs14993011

Gga_rs14055894

Gga_rs14058648

Gga_rs15696791

Gga_rs14059864

Gga_rs15709984

GGaluGA098404

Gga_rs15004928

GGaluGA102929

GGaluGA103021

Gga_rs15740759

GGaluGA105299

Gga_rs14087074

Gga_rs15772572

Gga_rs15775102

Gga_rs15026026

Gga_rs16731674

Gga_rs14102617

Gga_rs14102462

Gga_rs3137586

Gga_rs15028604

Gga_rs14097748

Gga_rs15037166

GGaluGA119889

Gga_rs15825243

GGaluGA122787

Gga_rs14416820

Gga_rs14116555

Gga_rs15853411

Gga_rs14274610

Gga_rs16169116

GGaluGA180722

Gga_rs14279037

GGaluGA181499

GGaluGA181650

Gga_rs15179975

Gga_rs13603297

Gga_rs16183652

GGaluGA186139

Gga_rs16184894

GGaluGA188072

GGaluGA188417

Gga_rs16190856

GGaluGA192373

GGaluGA193359

GGaluGA193688

Gga_rs16063926

GGaluGA195304

Gga_rs16201570

Gga_rs14300698

GGaluGA197809

Gga_rs14301381

Gga_rs14687412

GGaluGA200752

Gga_rs15245830

GGaluGA201319

Gga_rs14305761

Gga_rs16211508

GGaluGA202630

Gga_rs14707836

GGaluGA347845

GGaluGA348066

Gga_rs16103565

Gga_rs14757667

Gga_rs16760915

Gga_rs14755680

Gga_rs13788512

Gga_rs16764419

Gga_rs16764107

Gga_rs14707386

Gga_rs14759857

Gga_rs16105822

Gga_rs14687402

GGaluGA351173

Gga_rs16113312

Gga_rs14769351

Gga_rs16116824

GGaluGA354175

Gga_rs14731651

Gga_rs16774647

Gga_rs16121124

Gga_rs13802633

Gga_rs14781266

Gga_rs15990597
